# Kinase Fusions in Spitz Melanocytic Tumors: The Past, the Present, and the Future

**DOI:** 10.3390/dermatopathology11010010

**Published:** 2024-02-14

**Authors:** Maged Daruish, Francesca Ambrogio, Anna Colagrande, Andrea Marzullo, Rita Alaggio, Irma Trilli, Giuseppe Ingravallo, Gerardo Cazzato

**Affiliations:** 1 Dorset County Hospital NHS Foundation Trust, Dorchester DT1 2JY, UK; 2 Section of Dermatology and Venereology, Department of Precision and Regenerative Medicine and Ionian Area (DiMePRe-J), University of Bari “Aldo Moro”, 70124 Bari, Italy; francesca.ambrogio@policlinico.ba.it; 3 Section of Molecular Pathology, Department of Precision and Regenerative Medicine and Ionian Area (DiMePRe-J), University of Bari “Aldo Moro”, 70124 Bari, Italy; anna.colagrande@policlinico.ba.it (A.C.); andrea.marzullo@uniba.it (A.M.); giuseppe.ingravallo@uniba.it (G.I.); 4 Pathology Unit, Department of Laboratories, Bambino Gesù Children’s Hospital, IRCCS, 00165 Rome, Italy; rita.alaggio@uniroma1.it; 5 Department of Interdisciplinary Medicine, University of Bari “Aldo Moro”, 70124 Bari, Italy; irma.trilli@uniba.it

**Keywords:** kinase fusions, Spitz spectrum, melanocytic proliferations, ALK, ROS1, NTRK, MET, RET, MAP3K8, gene fusions

## Abstract

In recent years, particular interest has developed in molecular biology applied to the field of dermatopathology, with a focus on nevi of the Spitz spectrum. From 2014 onwards, an increasing number of papers have been published to classify, stratify, and correctly frame molecular alterations, including kinase fusions. In this paper, we try to synthesize the knowledge gained in this area so far. In December 2023, we searched Medline and Scopus for case reports and case series, narrative and systematic reviews, meta-analyses, observational studies—either longitudinal or historical, case series, and case reports published in English in the last 15 years using the keywords spitzoid neoplasms, kinase fusions, ALK, ROS1, NTRK (1-2-3), MET, RET, MAP3K8, and RAF1. ALK-rearranged Spitz tumors and ROS-1-rearranged tumors are among the most studied and characterized entities in the literature, in an attempt (although not always successful) to correlate histopathological features with the probable molecular driver alteration. NTRK-, RET-, and MET-rearranged Spitz tumors present another studied and characterized entity, with several rearrangements described but as of yet incomplete information about their prognostic significance. Furthermore, although rarer, rearrangements of serine–threonine kinases such as BRAF, RAF1, and MAP3K8 have also been described, but more cases with more detailed information about possible histopathological alterations, mechanisms of etiopathogenesis, and also prognosis are needed. The knowledge of molecular drivers is of great interest in the field of melanocytic diagnostics, and it is important to consider that in addition to immunohistochemistry, molecular techniques such as FISH, PCR, and/or NGS are essential to confirm and classify the different patterns of mutation. Future studies with large case series and molecular sequencing techniques are needed to allow for a more complete and comprehensive understanding of the role of fusion kinases in the spitzoid tumor family.

## 1. Introduction

Spitz neoplasms have always been the subject of heated debate among dermatologists and dermatopathologists due to their distinctive morphological, immunohistochemical, and molecular features [1]. Indeed, it has been one of the ‘grey areas’ of dermatopathology due to the low inter-observer agreement in classifying the melanocytic lesions of the Spitz spectrum, ranging from the frankly benign Spitz naevus (SN) to neoplasms with uncertain malignancy potential (atypical Spitz tumor, AST/melanocytoma), and the spitzoid melanoma (today better defined as Spitz melanoma, SM) [2]. In 2014, a pioneering paper by Wiesner and colleagues documented for the first time the presence of a kinase fusion in approximately half of all spitzoid neoplasms investigated [3] using targeted massively parallel DNA sequencing and laid the groundwork for further studies that detailed the presence of different gene rearrangements in this cohort of lesions. Today, as well as having understood that some Spitz lesions present a mutation for HRAS, a rather large number of fusion kinase genes have been described in the literature, and it has been demonstrated that Spitz neoplasms (including both nevi and atypical Spitz tumors, according to the current WHO classification of skin tumors) [3,4] with documented and confirmed translocation tend to occur in younger patients than neoplasms without gene rearrangements [5]. In addition, several studies have attempted to correlate the histological features to the type of rearrangement identified, demonstrating that morphology may be predictive of the recurrent molecular alteration in a subset of Spitz lesions [5]. It is also important to emphasize that the focus has been on kinase fusions as oncogenic driver mutations, as they are considered early events in the biological determinism of Spitz neoplasms, whereas the potential presence of such rearrangements in the continuation of the ‘biological history’ of the neoplasm (non-driver mutations) risks confusing assessments as they are considered stochastic events following but not responsible for the rearrangement of interest [3].

This new field of dermatopathology is of extraordinary importance for two main reasons: (1) these lesions, mainly AST, have uncertain biological behavior, and being able to subclassify them could lead to future risk stratification useful for the clinical and surgical management of these, often, young patients, and (2) frequently, these lesions can localize to loco-regional lymph nodes and be a cause for concern as part of the natural history of the pathology. In addition, further research in this area could also allow for the use of molecules that block these signal transduction pathways, such as Crizotinib in the case of ALK and ROS1 involvement.

In this review, we address the recent topic of kinase fusions in the spectrum of Spitz melanocytic proliferations, with a focus on the state of the art, emerging questions seeking answers, and future perspectives.

## 2. Materials and Methods

In December 2023, we searched Medline and Scopus for case reports and case series, narrative and systematic reviews, meta-analyses, observational studies—either longitudinal or historical, case series, and case reports published in English in the last 15 years using the keywords spitzoid neoplasms, kinase fusions, ALK, ROS1, NTRK (1-2-3), MET, RET, MAP3K8, and RAF1.

## 3. Results

### 3.1. ALK-Rearranged Spitz Tumors

Historically, anaplastic lymphoma kinase (ALK) has been studied in several cancer types, including anaplastic large-cell lymphoma, adenocarcinoma of the lung [6], epithelioid fibrous histiocytoma [7], and inflammatory myofibroblastic tumor (IMT) [8]. In the above-mentioned study [3], ALK rearrangement was found in 10–17% of Spitz tumors, most frequently in benign SNs and ASTs and less commonly in SM. Different fusion patterns linking the kinase domain of ALK to an N-terminal partner gene have been described in the literature; among these, the most common fusion genes involved tropomyosin 3 (TPM3) and dynactin 1 (DCTN1) [3,9], along with other reported genes such as GTF3C2, TPR, CLIP1, and NPM1 [10,11]. In terms of pathogenesis, the chimeric protein resulting from the fusion gene causes an increase in downstream phosphorylation and thus results in the activation of the MAPK and PI3K/AKT/mTOR pathway, which is upregulated and can be inhibited through the use of ALK inhibitors such as Crizotinib [3]. ALK-rearranged spitzoid tumors are the best characterized type in the literature, with rather clear clinical and histopathological data. These tumors have been reported to occur in patients ranging from 5 months to 64 years of age [5], but it is unclear whether there is a gender predominance [9,10]. Clinically, ALK-rearranged Spitz tumors frequently involve the lower limbs, are often amelanotic, and, for this reason, may be suspected to represent a vascular lesion (hemangioma, etc.), a keloid lesion, a verrucous lesion, xanthogranuloma, or even a melanocytic naevus [9,10].

From a histopathological point of view, these lesions are exophytic polypoid compounds, with a frequent plexiform growth pattern, with radially arranged fascicles of melanocytes with convergence towards the base in the dermis [3,5]. Although the wedge-shaped base appearance with an infiltrative border at the periphery is common, a bulbous to nodular ‘dumbbell’ growth pattern has rarely been reported [9,10]. Cytologically, melanocytes are plump and fusiform (spindled) rather than epithelioid, and the nuclei generally appear blunt and round in outline, with distinct nucleoli. In the various cases described in the literature, more than half had scattered intra- and perilesional lymphocytic infiltrate with sparse mitotic activity [10,12]. A recent paper [12] reported the case of a 3-year-old female with an exophytic papule on the vermilion border of her lower lip that had the morphological and immunophenotypical features of an ALK-rearranged Spitz tumor but was distinguished by its diffuse neurotropism (numerous nerve structures surrounded and/or expanded by the melanocytic proliferation), although in the absence of connotation of malignancy. The authors performed MelanoSITE FISH for the four interrogated loci, including RREB1 (6p25), cMYC (8q24), CDKN2A(p16)/CEN9, and CCND1(11q13), which were found to be within the normal limits.

Immunohistochemistry plays a major role in the correct screening evaluation of lesions that are morphologically suggestive of an ALK-rearranged Spitz tumor. When positive, the staining is intense and diffuse cytoplasmic and correlates well with molecular data [13] that can be obtained through fluorescence in situ hybridization (FISH), polymerase chain reaction (PCR), or next-generation sequencing (NGS) [3,5]. A paper [14] analyzed the prevalence of ALK gene alterations in a cohort of Spitz plexiform lesions, and 78 lesions were studied including 41 SNs, 29 ASTs, and 8 SMs. The authors used monoclonal antibodies for ALK in immunohistochemistry and FISH as confirmatory molecular data. Of their population of studied lesions, 14.6% (6 of 41) of the SNs expressed ALK, 13.8% (4 of 29) of the ASTs were positive for ALK, and none of the SM lesions expressed ALK. FISH confirmed translocation in nine cases and amplification involving the gene in one case. This work showed that the prevalence of ALK rearrangement was rather frequent when certain morphological features were present. Finally, it is important to emphasize that in some papers, the ALK immunostaining pattern is described as membranous with different types of fusion genes [15].

Although there are limited data in the literature, there has not yet been any reported recurrence or death in patients with an ALK-rearranged Spitz tumor, and the available sentinel lymph node biopsy (SLN) data have never demonstrated metastatic disease [3,5].

### 3.2. ROS1-Rearranged Spitz Tumors

ROS1 encodes a receptor tyrosine kinase of the insulin receptor family and is located on chromosome 6q22 [5]; fusions involving ROS1 have been reported in lung adenocarcinoma, glioblastoma, IMT, and cholangiocarcinoma [16]. In the paper by Weisner et al. [3], ROS1 fusions were detected in 17% of spitzoid neoplasms, more commonly in benign SNs (25%) than in ASTs/melanocytomas or Spitz melanoma. Different fusion gene partners (PWWP2A–ROS1, TPM3–ROS1, PPFIBP1–ROS1, MYH9–ROS1, CAPRINI1–ROS1, and MYO5A–ROS1) are reported in the literature and, in cell lines, it is noted that the fusion construct shows constitutively increased phosphorylation activity, with increased activation of downstream MAPK and PI3K signaling cascades [3,5]. There have been few papers that have attempted to correlate the clinical–morphological features of ROS1 rearranged tumors but it appears that there are some cases that can predict ROS1 translocation, compared to a subset of cases that have no specific architectural/cytological features. Gerami et al. reported the largest series of ROS1-rearranged Spitz tumors, consisting of ten SNs and seven spitzoid tumors with clinical, pathological, and molecular correlations [17]. The age of the patients ranged from 3 to 58 years, with a mean age of 19 years; ten patients were female and seven patients were male. Four lesions were located in the head/neck region, three were located in the upper extremities, three were located in the trunk, and seven were located in the lower extremities. Clinically, the lesions presented as pink to red papules with clinical suspicion of atypical Spitz naevus (8), dermatofibroma (2), benign naevus (2), pyogenic granuloma (2), and a cyst (1). Follow-up data were available in 13 cases (13/17) with a mean follow-up of 23 months (4–95 months). The silhouette was plaque-like in seven cases, nodular in seven cases, wedge-shaped in two cases, and polypoid in one case. Histologically, 12 cases showed small to intermediate epithelioid- and spindle-shaped cells with floating nests in the epidermis with trans-epidermal elimination of nests and pagetoid spread. In four cases, the spindle cell component was prominent. Mild/moderate nuclear atypia was present in all lesions, while a regular maturation gradient, a low mitotic index of 1.3/mm^2^, and Kamino bodies were also seen.

The fusion partner was identified in 16 of the 17 cases in the study and the most common genomic fusions were a *PWWP2A::ROS1* fusion, seen in 6 cases, and a *TPM3::ROS1* fusion, seen in 5 cases. Other recurrent fusion partners included *PPFIBP*, in two cases, and *MYH9*, *CAPRINI1*, and *MYO5A*, each seen in one case.

From an immunohistochemical point of view, staining with the anti-ROS1 monoclonal antibody is generally weak and, the rearrangement should be confirmed using FISH, PCR, or NGS [5,18].

### 3.3. NTRK-Rearranged Spitz Tumors

The NTRK1 gene (1q), NTRK2 gene (9q), and NTRK3 gene (15q) encode the transmembrane receptors TrkA, TrkB, and TrkC, respectively [19], with all of them being involved in the response of melanocytes to neurotrophic signals [20]. Trk stimulation results in proliferation and migration in melanoma cell lines [21]. Translocations involving the NTRK genes result in overactivation or overexpression of the Trk receptors [22], with subsequent downstream activation of MAPK, PI3-K, and PLCg pathways [20]. NTRK rearrangements were identified in spindle cell mesenchymal neoplasms and included as a provisional entity in the fifth WHO classification of soft tissue tumors [4].

#### 3.3.1. NTRK1-Rearranged Spitz Tumors

NTRK1 fusions have been found across the entire Spitz spectrum including SNs, AST/melanocytoma, and SM [3,23], with them representing 23% of cases overall and having high prevalence in both of the latter groups [3,24,25]. Reported partner genes include LMNA, TPM3, TP53, and KHDRBS1 [25]. Certain morphologic features have been associated with NTRK1-fused Spitz tumors, including the filigree-like appearance of the rete ridges, lobulated nests of melanocytes with rosette-like structures, and prominent maturation of tumor cells with depth [19,25,26]; however, these features are only seen in a minority of cases and, hence, their absence does not rule out an NTRK fusion [27]. Spitz harboring NTRK1 rearrangement may also show a plexiform pattern similar to that seen in ALK Spitz lesions [28]. Kamino bodies are variable, and approximately 25% of cases will have a degree of pagetoid spread [29]. Both Pan-trk and NTRK1 are reliable and cost-effective but non-specific methods for screening for NTRK fusion [23,27,28,29,30]. 

#### 3.3.2. NTRK2-Rearranged Spitz Tumors

Few Spitz tumors with NTRK2 rearrangements have been reported after the description of the first lesion with an NTRK2::TGF fusion, identified in a pigmented spindle cell naevus (PSCN) of Reed, with evident Kamino bodies [31]. More recently, five cases have been described with an NTRK2 fusion with the SQSTM1 gene (5q), whose protein product is involved in NF-kB signaling, apoptosis, and autophagy [32]. These tumors share a polygonal morphology of tumor cells with dendritic processes, prominent lentiginous patterns with involvement of the hair follicle epithelium, and small hyaline structures which are likely incipient Kamino bodies [32].

#### 3.3.3. NTRK3-Rearranged Spitz Tumors

NTRK3 fusions can be detected in 0.7% of Spitz tumors, and the most frequent fusions include ETV6::NTRK3, MYO5A::NTRK3, and MYH9::NTRK3 [33]. ETV6::NTRK3 fusion has been identified in not only infantile mesenchymal neoplasms, such as infantile fibrosarcoma/congenital “cellular” mesoblastic nephroma and secretory carcinomas of the breast, salivary gland, skin [34,35,36,37], but also in thyroid cancers associated with a history of radiation [38]. ETV6::NTRK3 fusion is also reported in acute myelogenous leukemia, chronic eosinophilic leukemia, inflammatory myofibroblastic tumor, pediatric high-grade glioma, and metastatic colorectal carcinoma [39,40,41,42,43]. In one study, 57% of the PSCN of Reed had NTRK3 fusions, with MYO5A being the most identified partner. The presence of the NTRK3 fusion is significantly associated with the younger age of patients and the involvement of the adnexa [44]. 

The fusion partner was found to influence the NTRK3 fused Spitz morphology. ETV6::NTRK rearranged lesions show monomorphic epithelioid melanocytes with distinct cell borders and abundant pale cytoplasm. Lobulated coalescing nests similar to those seen in NTRK1 fusion cases may be seen. ETV6-rearranged Spitz tumors tend to be seen in younger patients and localize on the face [45]. Those with MYO5A::NTRK3 fusions show spindle cells arranged in a fascicular pattern with neuroid features and Verocay’s body-like appearance [45]. MYO5A encodes myosin V protein, which plays a role in the transportation of melanosomes [46]. Autosomal recessive loss of function of MYO5A and subsequent pigment dilution is characteristic of Griscelli syndrome [47]. 

Melanomas harboring pan-NTRK fusion appear to have an indolent behavior with only rare lymph node metastasis [48,49]. PanTrk IHC pattern of staining may be predictive of the fusion partner, as intense nuclear staining is seen with ETV6::NTRK3 fusion, while linear staining is indicative of MYO5A::NTRK3 fusions [50].

### 3.4. RET-Rearranged Spitz Tumors

RET fusion has been identified in 3–4% of Spitz tumors [4,51]. In addition, it has been described as an oncogenic driver in papillary thyroid carcinoma, lung carcinomas, and salivary, breast, and colorectal carcinomas [52,53,54,55,56]. Spindle cell proliferations harboring RET fusion with morphologic similarity to the NTRK fused tumors have been reported [57]. The Ret proto-oncogene, located on chromosome 10q, encodes a tyrosine kinase protein with roles in cell proliferation, migration, and differentiation [58]. Activation of the RET kinase domain through structural rearrangements results in the activation of various pathways such as the MAPK, PI3K/AKT, and PLCg1 pathways. Known gene fusion partners include CCD6, KIF5B, LMNA, GOLGA5, and MYO5A [4,18,58]. While no specific histopathological features for RET Spitz tumors have been established, a 2021 study described a plaque-like silhouette in five Spitz neoplasms and a monotonous morphology with predominantly epithelioid melanocytes. Dyscohesion of the tumor cells within the nests was noted in thre of the five cases [58]. RET fusion ASTs and SM appear to have a favorable outcome with no reported distal metastasis or deaths [4,18,58].

### 3.5. MET-Rearranged Spitz Tumors

MET kinase fusion was not identified in the original study, and reports remain extremely sparse in the literature, with it representing approximately 0.5% of all driven melanocytic lesions [23,59]. MET is a proto-oncogene on chromosome 7q that encodes a tyrosine kinase receptor with roles in angiogenesis and cellular growth as well as melanocyte development [5]. Identified fusion partners to date include TRIM4, ZKSCAN1, LRRFIP1, PPFIBP1, EPS15, and DCTN1 [12], and the resultant fusion protein led to activation of the MAPK, PI3K/AKT, and PLCg1 pathways [59]. On a morphological level, due to their rarity, no unique associated histopathological features have been recognized, with tumors showing the classical Spitz appearance, including large nests of epithelioid/spindled melanocytes and epidermal hyperplasia [27,59]. MET immunohistochemistry, while non-specific, has shown strong and diffuse cytoplasmic positivity in three tested cases in comparison to conventional naevi [46]. Molecular detection modalities include FISH, NGS, and RT-PCR. No adverse outcomes have been reported in that cases of AST and SM harboring the fusion with long-term follow-up [27,59].

### 3.6. Serine–Threonine Kinase-Rearranged Spitz Tumors

#### 3.6.1. BRAF-Rearranged Spitz Tumors

The BRAF gene encodes a RAF kinase which activates the MAPK pathway through downstream RAS signaling. BRAF functions relate to cell division and differentiation, and BRAF mutations (most commonly at the V600 amino acid) lead to uncontrolled cell division [60]. BRAF mutations are characteristic of ordinary naevi and melanomas. Whether or not the detection of a BRAF mutation excludes a diagnosis of a Spitz lineage remains controversial, especially with the recent description of BRAF mutated and morphologically spitzoid nevi and tumors (BAMS) [61]. On the other hand, BRAF structural rearrangements as drivers for Spitz lineage are well established. Approximately 5–5.7% of Spitz lesions harbor a BRAF fusion [23,51]. It has also been identified in various solid neoplasms including thyroid cancer, gliomas, pancreatic cancer, and non-small-cell lung cancer [59]. 

Overall, BRAF fusions appear to significantly correlate with desmoplastic stroma and epithelioid melanocytic morphology [25,62,63,64,65], although a spindle cell morphology has also been reported [62]. Identified fusion partners include AKAP9, CLIP2, SKAP2, AGK3, MYO5A, and MLANA [63,64,65]. The melanocytes can be arranged within the stroma as single cells with buckshot appearance or in sheets [26,63]. Polypoid, plaque, and nodular silhouettes have been observed [26,62,63]. Interestingly, multiple BRAF fusion naevi with desmoplasia were reported in one patient with ring chromosome 7 syndrome [66]. BRAF fused tumors appear to have more frequent nuclear atypia and pleomorphism, and SMs with BRAF fusions can behave in a more aggressive fashion, with reported distant metastasis in four cases [64]. In one paper [49], a case of death in a case of BRAF fusion Spitz tumor is reported.

#### 3.6.2. RAF1-Rearranged Spitz Tumors

Most recently, three cases of Spitz neoplasms, 2 Spitz naevi, and 1 Spitz melanoma have been reported with AN RAF1 rearrangement as the driver of molecular aberration [67]. The partner genes included CTDSPL, PPAP2B, and ATP2B4. No specific morphological features have been recognized [67]. The RAF1 gene (3p) encodes CRAF, a serine–threonine protein kinase that acts by forming a dimer with BRAF and activating the MEK-ERK pathway with resultant cellular proliferation [68,69]. Other reported melanocytic tumors involving fusions of RAF1 include malignant melanoma, BAP1-inactivating melanocytoma, and congenital naevi [68,70,71,72].

#### 3.6.3. MAP3K8-Rearranged Spitz Tumors

Rearrangements involving the MAP3K8 oncogene have been identified as the driving tumorigenic alteration across the Spitz spectrum [43,73,74,75]. MAP3K8, which encodes a serine–threonine kinase, was first recognized as an oncogene in thyroid cancer [76]. It has been since found in squamous cell carcinoma, keratoacanthoma, and ovarian carcinoma [77,78]. In the setting of Spitz lesions, numerous partner genes have been reported, of which Supervillin (SVIL) appears to be the most common [73,74]. Spitz neoplasms with MAP3K8 fusion have epithelioid melanocytes with variable pigmentation and usually high-grade cytological atypia with common giant multinucleated cells [28,74,75,76]. Indeed, MAP3K8 fusion has a high prevalence in lesions classified as ASTs/SMs [28,75]. These tend to show epidermal ulceration, deep mitotic figures, and inactivation of CDKN2A that can be detected through immunostaining for p16 or FISH [28,74,75,76,77,78]. Positive sentinel lymph nodes have been reported [73].

Table 1 summarizes the clinical, histopathological, and molecular features of kinase fusions rearranged-Spitz tumors.

**Figure 1:**
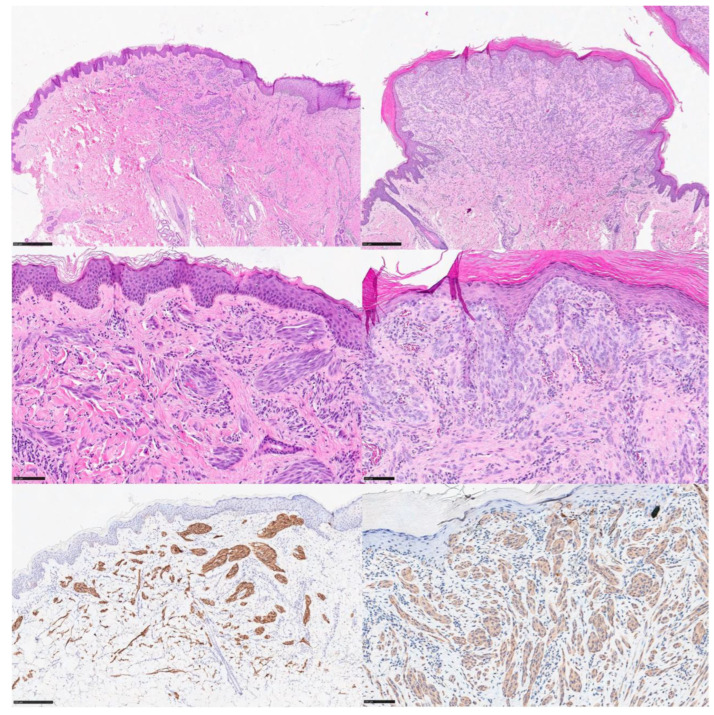
**Left side**: An example of ALK-rearranged Spitz nevus characterized by a plexiform pattern with cytological features of Spitz lineage (nucleoli with abundant eosinophilic cytoplasm) and diffuse, strong cytoplasmic immunostaining with the ALK 01 antibody. **Right side**: An example of ROS1-rearranged Spitz nevus characterized by a nodular growth pattern with cytological features of Spitz lineage; note the weak but diffuse positivity for the ROS1 antibody.

Figure 1 represents some examples of ALK- and ROS1-rearranged spitz tumors.

## 4. Conclusions and Future Directions

Despite the recent advances in the detection of driving molecular alterations in Spitz lesions, a significant proportion lack a detectable driver and need further exploration. Morphological features, while not always specific, may help predict the driving molecular aberrations and subsequently, targeted immunohistochemical or genetic studies. The molecular alteration can be further confirmed by molecular methods such as FISH, PCR, and/or NGS. 

Interestingly, Spitz tumors with serine–threonine kinase fusions (particularly involving BRAF and MAP3K8 genes) are prevalent among the AST/SM end of the spectrum. One theory is that these lesions may have genome instability and are more prone to progress to malignancy through acquiring further genetic aberrations such as copy number changes or CDKN2A inactivation, although the true behavior of these neoplasms is a question that remains to be answered. Regarding the prognostic significance of these kinase fusions, the vast majority of reported cases have not exhibited aggressive behavior, postponing the answer to what the clinical impact of these mutations may be.

Future studies with large case series and molecular sequencing techniques are needed to allow for a more complete and comprehensive understanding of the role of fusion kinases in the spitzoid tumor family.

## Figures and Tables

**Table 1:** Clinical, histopathological, and molecular features of kinase fusion rearranged Spitz tumors such as ALK, ROS1, and NTRK.

Type of Kinase Fusions (Most Frequently Reported)	Clinical Features (Most Frequent, Rarely Present)	Histopathology (Usually, Rarely)	Molecular Features (Most Frequent Gene Patterns and Rare Gene Patterns)
ALK-rearranged	Age: 5 months–64 years of age Lower limbs Amelanotic	Exophytic polypoid compound lesions Sometimes plexiform Bulbous to nodular growth	TPM3 DCTN1 GTF3C2, TPR, CLIP1 and NPM1
ROS1-rearranged	Age: 3–58 years Head/neck, upper extremities, trunk, and lower extremities Red papules	Plaque-like Nodular Wedge-shaped Polypoid	PWWP2A, TPM3, PPFIBP1 MYH9, CAPRINI1, and MYO5A
NTRK-rearranged	Wide distribution of age and topography Younger patients and localization on the face	NTRK1: Filigree-like appearance of rete ridges and lobulated nests of melanocytes Sometimes plexiform patterns	NTRK1: LMNA, TPM3, TP53, and KHDRBS1
		NTRK2: Polygonal morphology of tumor cells with dendritic processes Prominent lentiginous pattern Incipient Kamino bodies	NTRK2: TGF and SQSTM1
		NTRK3: Monomorphic epithelioid melanocytes with distinct cell borders and abundant pale cytoplasm	NTRK3: ETV6, MYO5A, and MYH9

Legend: ALK: anaplastic lymphoma kinase; TPM3: tropomyosin 3; DCTN1: dynactin 1; GTF3C2: general transcription factor IIIC subunit 2; TPR: translocated promoter region; CLIP1: CAP-Gly domain-containing linker protein 1; NPM1: nucleophosmin 1; PWWP2A: PWWP domain-containing protein 2A; PPFIBP1: PPFIA binding protein 1; MYH9: myosin heavy chain 9; CAPRINI1: cell cycle-associated protein 1; MYO5A: myosin VA; LMNA: lamin A/C; KHDRBS1: KH RNA binding domain-containing, signal transduction-associated 1; TGF: transforming growth factor; SQSTM1: sequestosome 1; ETV6: ETS variant transcription factor 6.
